# The Relationship of Acculturation Strategies to Resilience: The Moderating Impact of Social Support among Qiang Ethnicity following the 2008 Chinese Earthquake

**DOI:** 10.1371/journal.pone.0164484

**Published:** 2016-10-14

**Authors:** Li Han, John W. Berry, Yong Zheng

**Affiliations:** 1 Mianyang Normal University, Mianyang, China; 2 Queen’s University, Kingston, Canada; 3 National Research University Higher School of Economics, Moscow, Russia; 4 Center for Studies of Education and Psychology of Ethnic Minorities in Southwest China, Southwest University, Chongqing, China; Western Sydney University, AUSTRALIA

## Abstract

International research has mostly confirmed the positive association between acculturation strategies and resilience in ethnic groups, but the mediating and moderating mechanisms underlying the relationships are still under-investigated. The present study aimed to investigate the associations between acculturation strategies (based on two cultural identities) and resilience of 898 Qiang ethnicity volunteers (mean age = 29.5), especially exploring the mediating and moderating effects of personality, spiritual belief and social support on the relationship between acculturation strategy (using two cultural identities as latent variables in model analysis) and resilience following the occurrence of 2008 Wenchuan earthquake in Sichuan, taking such mechanisms into account. Results were as follows: (1) All variable presented significant positive correlations; (2) Consistent with the mediating hypotheses, personality and spiritual beliefs played a partial mediating role in the relationship between two cultural identities and resilience; (3) High or low level of perceived social support had a moderating effect on cultural identities and resilience; (4) The integration strategy was the most optimal style to promote the development of resilience, but marginalization was the least effective style.

## Introduction

An earthquake of magnitude 8 struck Sichuan province in China on May 12, 2008. The catastrophic event caused 69,142 deaths with 17,551 people missing [1]. The disaster was most severe in areas where majority of people of Qiang ethnicity resided: Wenchuan, Beichuan, Mao county, Li county, and Pingwu. The disaster caused a range of negative psychological consequences among survivors, such as anxiety, depression and posttraumatic stress disorder (PTSD) [2–4].

Numerous studies have concluded that resilience protects against certain negative psychological consequences, such as PTSD [5] and depression [6]. Resilience has been shown to contribute to disaster survivors’ recovery [7], and is usually positively related to individual’s mental health and quality of life [8]. There are fifty-five ethnicities in China from. They have different cultural backgrounds, which, had come to live together in this diverse society, gradually forming a plural multicultural society [9, 10]. In these culture-contact settings, acculturation plays an important role in determining how well people adapt both psychologically and socio-culturally [11]. Acculturation, refers to the ways in which members of ethnocultural groups undergo a change following intercultural contact, and become involved in the larger society in which they reside [12]. Some acculturation scholars have asserted that the principles of acculturation theory are grounded in the broader psychological theory of stress and coping [13, 14]. A resilience-based model of acculturation found that acculturation hassles have both direct and indirect effects on negative affect and have an indirect effect on positive affect. Specifically, threat appraisal and sense-making coping have a partial mediation effect on acculturation hassles and negative effect, while the effect of acculturation hassles on positive affect is mediated by threat appraisal, sense-making coping and meaning-in-life [15]. The protective factor model of resilience also suggested that, in relation to low levels of protective factor, higher levels of protective factor buffer the relation between the risk factor and adjustment [16, 17]. Over the past several years, positive psychology research has focused on relationships between individual positive psychology variables and outcomes. However, only a few studies have examined the relationships between multiple positive psychology variables such as resilience, social support, and spirituality/religion and their differential influence on mental health outcomes. Therefore, the aim of the current study was to examine the relationships between acculturation strategies and resilience, taking into account the mediating role of other protective factors of resilience such as spiritual belief (defined as a positive sense of meaning and purpose in life, such as supernatural belief, social belief, and pragmatic belief), personality(defined as individual characteristics that account for consistent behavioral patterns showed as extraversion, neuroticism, openness, conscientiousness, and agreeableness), and social support (defined as the perceived availability of resources provided by government, organization, family, friends and peers that assist the person in everyday activities) on people of Qiang ethnicity following the 2008 Chinese earthquake.

### Acculturation

Acculturation is broadly defined as a process of cultural and psychological change that occurs when two cultural groups interact [11]. Cultural changes include alterations in a group’s customs, and in their economic and political life. Psychological changes include alterations in individuals’ attitudes toward the acculturation process and their cultural identities. Acculturation has a strong conceptual and empirical appeal in psychological research because of its hypothesized, as well as demonstrated, relationships to a wide array of psychosocial factors among ethnic groups that are in culture contact settings. These include: mental health [18], sociocultural adaptation [14], acculturative stress [19, 20], self-identity, and personality [21], to name a few. Berry [12, 22] proposed a bi-dimensional acculturation model based on two issues important to those who are in intercultural contact. These issues refer to the extent to which individuals and groups (1) seek to maintain their heritage culture and identity; and (2) seek to have interactions with people of other cultures in the larger plural society. When these two dimensions are crossed, four acculturation strategies are defined: assimilation, separation, integration, and marginalization. Assimilation exists when individuals do not wish to maintain their heritage culture, and seek to become fully involved with the larger society; Separation exists when ethnic people place a value on holding on to their original culture, and at the same time, wish to avoid interaction with the dominant culture. Integration exists when individuals wish to maintain their heritage culture and also aspire to be fully engaged in the life of the larger society. Marginalization, an exact opposite of integration, reflects minimal interest in either heritage cultural maintenance or connection with dominant culture. A number of studies have assessed the association between acculturation strategies and psychological outcomes following a traumatic event [23, 24]. Some suggest that low assimilation is associated with poorer health outcomes [25, 26], while others show no differences in health due to low assimilation or that low assimilation is associated with better outcomes [27, 28]. In the current study, we examined the effects of these four acculturation strategies on resilience. These four strategies are based on two Qiang cultural identities: their identity with their heritage culture (ethnic identity) and their identity with the larger National Chinese society (national identity).

### Resilience

Particularly during the last two decades, numerous researchers, clinicians, psychologists, and sociologists have shifted their focus from risk to resilience [29–32]. Although no universal definition of resilience has yet been established, it is frequently described by two theoretical perspectives: a personality trait and a process. As a trait, resilience is defined as a personal characteristic that allows for success in the face of adversity [33, 34]. As a process, resilience involves contextual, environmental, societal, and cultural aspects as well as relationships and opportunities that are available to individuals [35, 36]. Generally, resilience can be defined as reduced vulnerability to environmental risk experiences, the overcoming of a stress or adversity, or a relatively good outcome despite risk experiences [37]. For the current study, we defined resilience as a cluster of personality traits, which undergo a change along with personal growth [33].

These characteristics of resilience enable individuals to deal effectively with adversity. There is much evidence supporting that resilience might help to improve one’s well-being and promote recovery from stressful situations [38, 39]. Moreover, characteristics of resilience (e.g., tenacity, personal strength, and optimism) can alleviateindividual depressive symptoms following trauma events [16, 40]. Despite many studies on resilience over the past decade, much of the available research has focused on risk for poor outcome rather than resilience *per se*. [41, 42]. In the current study, we focus on predictors of resilience and on mediating and moderating effect paths.

### Acculturation, Personality, Spiritual Belief, and Social Support Effect on Resilience

A growing number of studies have assessed the association between acculturation and psychological outcomes following a traumatic event [43, 44]. Many empirical studies have emphasized the positive contributions of cultural identity and acculturation in promoting resilience among the youth from diverse cultural and ethnic backgrounds [45, 46]. It is widely assumed that resilience is determined to a large extent by personality [47]. Some studies have also demonstrated associations between personality variables and explicitly defined resilient outcome trajectories [48,49]. With reference to protective factors at the individual level, other factors that promote resilience include a person’s spirituality beliefs, religious assets, and social support. Valentine and Feinauer [50] noted several resiliency themes, one of which was spirituality. Spirituality has been shown to be a key in promoting resilience [51] and enhancing coping mechanisms in negative life events [52]. Higher levels of religious involvement are modestly associated with better health, after taking account of other influences, such as age, sex, and social support [53]. The social support systems are important protective factors for children and adolescents experiencing environmental hazards [54]. The buffering model of social support hypothesizes that social support protects individuals from the potentially harmful effects of stressful events [55]. Hence, in the current study, in addition to acculturation strategies, we considered personality and spiritual beliefs as mediators in the mixed model that attempts to predict resilience under the moderating effect by social support.

### The Present Study

The aim of the current study was to examine a structural model of resiliency in relation to four acculturation strategies (that were derived from crossing two cultural identities), personality, spiritual beliefs and social support among people of Qiang ethnicity in China. First, we examined the intercorrelation among all variables. Second, we attempted to construct a structural equation model for testing mediation and moderation effects between acculturation strategies (based on cultural identities) and resilience. Third, a one-way analysis of variance was conducted to observe the influence of acculturation strategies on resilience.

## Method

### Participants

Participants in the current study consisted of 898 Qiang ethnic volunteers who came from Aba Tibetan and Qiang Autonomous Prefecture of Sichuan province. Of 898 participants, 46.1% (*n* = 414) were male and 53.9% (*n* = 484) were female. The participants’ ages ranged from 18 to 68 years, with a mean age of 29.5 years (*SD* = 11.23). In terms of educational level, 55% of the respondents reported having a college degree, 32% of participants had middle school education level, and 13% of the participants were primary school graduates. Although Qiang people maintained their own cultural identity and cultural heritage, they integrated into the larger society relatively well. Most of the Qiang people were employed in various economic sectors such as education, agriculture, industries, and commercial business. This study was approved by the ethics committee of the Faculty of Psychology at the Southwest University, China. Written informed consent was obtained from each participant before the commencement of the study.

### Measures

Qiang Acculturation Questionnaire (QAQ). To assess the acculturation of Qiang people in Chinese society, we developed the QAQ [56]. There were 47 items, with participants responding on a five point scale (0 = not true at all, 4 = true nearly all the time). Higher scores indicated more acculturation on two dimensions of acculturation framework: *Qiang cultural identity* (QCI) and *National cultural identity* (NCI). Factor analysis of 30 items in the QCI revealed three dimensions: Qiang knowledge and behavior, Qiang religious identity, and Qiang pride. The Cronbach’s alpha coefficient of QCI was 0.92. NCI was assessed with 17 items. Factor analysis showed that this identity also had three components: National cultural knowledge, National symbolic beliefs, and National customs. Cronbach’s alpha coefficient of NCI was 0.88.

The NEO Personality Inventory (NEO-PI-R) [57] is a widely used instrument to assess personality variables. The Revised Chinese version of the NEO-PI-R consists of 60 items which assess the personality dimensions through 12 traits with a list of descriptive attributes [58]. These 12 traits are supposed to cluster into the 5 factors (extraversion, neuroticism, openness, conscientiousness and agreeableness). Each trait consists of several items asking participants to indicate to what extent each item fits their condition (1 = not at all; 4 = a lot). Higher scores on each construct indicate higher levels of that trait. The internal consistency reliability coefficients (Cronbach-alpha) of the scale is 0.88.

Connor-Davidson Resilience Scale (CD-RISC) [33] is a 25-item measure influenced by Kobasa’s [59] work with hardiness. The Chinese version CD-RISC was revised by Yu and Zhang [60]. Respondents rated items on a scale from 0 to 4 (0 = not true at all, 4 = true nearly all the time). Higher scores indicated higher resilience. This study produced a three-factor structure—tenacity, strength, and optimism—which corresponded to certain features of Chinese culture. More notably, the Chinese version of CD-RISC was demonstrated to be a reliable measurement (Cronbach’s alpha = 0.89) in assessing resilience among Chinese sample after the 2008 earthquake [61]. In this study, the Cronbach’s alpha coefficient was 0.86.

Spiritual Belief Questionnaire (SBQ) compiled by Song and Yue [62] was used. It had 39 items, including 3 branch scales: supernatural belief, social belief, and pragmatic belief. Respondents rated items on a scale of one to five (1 = not true at all, 5 = true nearly all the time). The Cronbach-alpha of the whole scale was 0.68, and those of the three branch scales ranged from 0.66 to 0.84.

Perceived Social Support Scale (PSSS) is a 12-item measure compiled by Blumenthal et al. [63], and it was used for assessing social support. PSSS were assessed on 5-Liket score ranging from 1 = totally disagree, to 5 = totally agree, and higher scores indicated more perceived social support. Cronbach-alpha of the whole scale was 0.89, and those of the three subscales (e.g., support from family, support from friend, and support from significant others) ranged from 0.81 to 0.83.

Demographic Characteristics. We measured two demographic characteristics of Qiang people: age (young, 48.3%; adult, 51.7%); and education (primary school 13.3%; middle school 15.8%; high school 16.1%; university 54.8%).

### Statistical Analyses

The data analysis was carried out using SPSS 18.0. The measured model analyses were conducted using AMOS 20.0 (IBM, Inc), applying maximum likelihood estimation methods. The structural model was conducted to test the significance of the mediated and moderated effects of cultural identities on resilience. Fit statistics assessed included the root mean square error of approximation (RMSEA) as the primary fit criterion, with an RMSEA of 0.05–0.08 [64] or less an indication of excellent fit. And other model fit indices would be reported such as GFI, AGFI, NFI, CFI and IFI.

## Results and Analysis

### The Statistical analysis of Common Method Biases

Since data from self-reports may produce method biases, we adopted an anonymous survey and reversed some items in data processing [65]. After data collection, we examined the common method biases by the Harman [66] single factor test. The results indicated five-factor eigenvalues greater than one, and the first factor could explain the variance of 14.35% (which is less than 40% of the critical value). Thus, the threat of common method biases is not present in this study.

### Intercorrelations between all variables

Table 1 presents zero-order correlations and descriptive statistics among the primary variables. All variables presented significant positive correlations.

**Table 1:** Zero-order correlations, descriptive statistics among all variables (*N* = 898).

Variables	1	2	3	4	5	6	*M*	*SD*
1. Qiang cultural identity	—						3.22	0.96
2. National cultural identity	0.440^a^	—					3.63	0.92
3. Personality	0.224^a^	0.336 ^a^	—				3.15	0.22
4. Spiritual belief	0.338 ^a^	0.222 ^a^	0.190 ^a^	—			3.16	0.43
5. Social support	0.132 ^a^	0.317 ^a^	0.316 ^a^	0.301 ^a^	—		4.43	1.07
6. Resilience	0.304 ^a^	0.420 ^a^	0.494 ^a^	0.407 ^a^	0.301 ^a^	—	3.30	0.52

^a^ *p* < 0.01.

### A structural equation model of resilience among Qiang sample

**Fig 1:**
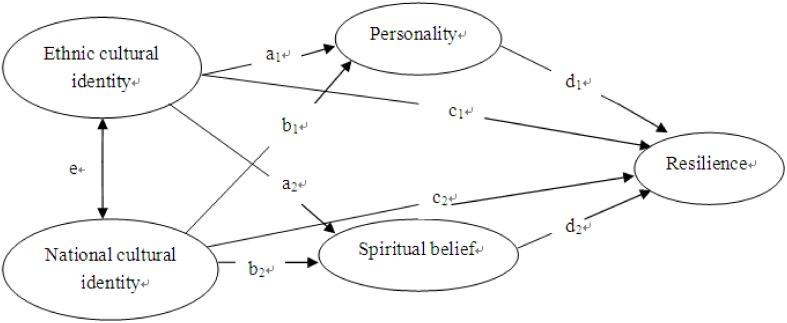
The conceptual model of resilience.

We proposed a conceptual model (Fig 1) based on a previous research on resilience. Personality and spiritual beliefs were used as mediating variables, and perceived social support (high or low) as the moderating variable. The three Qiang cultural identity factors formed one variable and three National cultural identity factors formed another.

**Fig 2:**
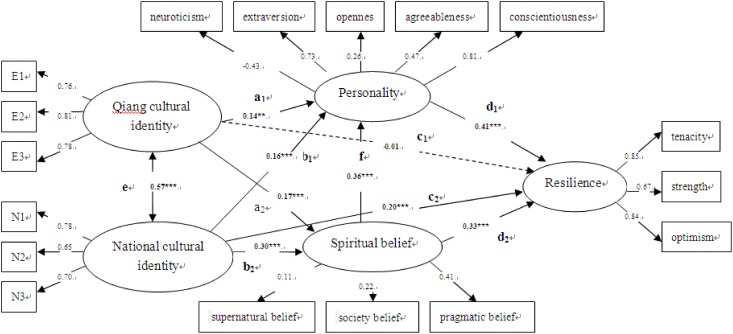
The modified model of resilience. Note. E1 = Qiang religious identity, E2 = Qiang pride, E3 = Qiang knowledge and behavior; N1 = National knowledge, N2 = National symbolic beliefs, N3 = National customs.

Since this model on resilience involved a combination of two cultural identities and personality trait and spiritual beliefs, overall this measurement model provided a good fit to the data (Fig 2; Table 2). Table 2 reports the standardized estimates for the structural models that were conducted to evaluate the mediation hypotheses. The standardized path estimates in Table 2 for the *a*_*1*_, *a*_*2*_, *b*_*1*_, *b*_*2*_, and *d*_*1*_, *d*_*2*_ paths corresponded to those illustrated in Fig 2 for each mediator and resilience analyzed separately. The c1,c2 paths represented the direct effects of cultural identities on resilience, separately, and *f* path indicated the direct effect of spiritual belief on personality in the modified model. In addition, there was a covariant relation between the two cultural identities.

**Table 2:** The mediation effect structural equation model of fit indices.

model	*χ*^2^	*df*	*χ*^2^ */df*	RMSEA	GFI	AGFI	NFI	CFI	IFI
initial model	724.842	104	6.970	0.082	0.910	0.868	0.901	0.914	0.914
modified model	360.089	94	3.831	0.052	0.956	0.928	0.951	0.963	0.963
validation model	389.053	94	4.139	0.056	0.920	0.896	0.921	0.935	0.935

Note. We randomly selected half of Qiang sample (*n* = 449) to test the initial model and modified model, and using other half test the validation model. *χ*^2^ */df =* normed chi-square; RMSEA = root mean square error of approximation; GFI = goodness-of–fit index; AGFI = adjusted goodness-of–fit index; NFI = normed fit index; CFI = comparative fit index; IFI = incremental fit index.

The mediation effect analysis (Fig 2) found that ethnic cultural identity prediction effect on resilience decreased from 0.15 (*p* < 0.001) to -0.01(see *c*_*1*_ path, *p* > 0.05), and ethnic cultural identity prediction effect on personality was 0.14 (see *a*_*1*_ path, *p* < 0.01) after adding the mediator variables (personality and spiritual belief). Thus, personality and spiritual belief played a complete mediation effect on ethnic cultural identity and resilience, and the effect size of 0.057. In the same way, the National cultural identity prediction effect on resilience also decreased from 0.40 to 0.20 (see *c*_*2*_ path, *p* < 0.001) after adding the mediation variables, and the mediator had a partial mediation effect on national cultural identity and resilience. The mediation effect size was 0.099, which accounted for an overall of 33.11%. Furthermore, the *f* path indicated that spiritual belief significantly affected personality, and the prediction effect size was 0.355. In addition, we compared modified model and validation model of resilience, and the results showed that most of the fit indices were very close. It was suggested that the modified model was supported by the other half of the total samples.

Table 3 shows the results of moderating effect between cultural identities and resilience through perceived social support. We examined two models using two levels of social support (high or low groups). One was default model (all the parameters had free estimation), the other was constraint model (the regression weights of parameters were equal). Grouping regression analysis showed that chi-square value changed significantly (*p* < 0.001). Therefore, social support remarkably moderated the latent variables in this model.

**Table 3:** The moderation effect structural equation model of fit indices.

	*df*	CMIN	*P*	NFI	IFI	RFI	TLI
				Delta-1	Delta-2	rho-1	rho-2
Constraint model	10	589.912	0.000	0.154	0.165	0.157	0.169

**Fig 3:**
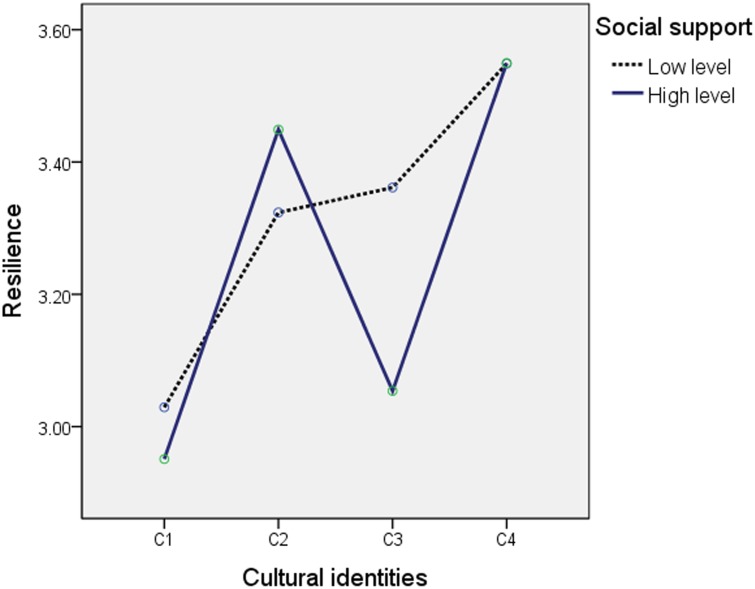
The interaction graph of resilience. Note. Qiang cultural identity = QCI, National cultural identity = NCI, C1 = Low QCI and Low NCI, C2 = High QCI and Low NCI, C3 = Low QCI and High NCI, C4 = High QCI and High NCI.

How does social support exert the moderating effect? We explored the interaction of different social support levels and cultural identities on resilience (Fig 3). The result showed that there was significant interaction effect, *F*(3, 486) = 2.857 (*p* < 0.01). That is, the four cultural identities had an effect on resilience with changes in high or low level of social support.

### One-way analysis on cultural identities and resilience for different acculturation strategies in Qiang sample

According to the acculturation strategies framework, there are four expected acculturation strategies. In this study, two cultural identities (the QCI and NCI scales) were used to create four acculturation strategies (integration, assimilation, separation, and marginalization) by dividing them at the mean of each scale. We found that there were 341 persons in the integration group, 270 in the marginalization group, 188 in the assimilation group, and 99 in the separation group. The resilience levels across the four acculturation strategies were significantly different (*p* < 0.001), and the effect size was at about medium level (*η*^2^ = 0.149–0.200). Qiang people who used the integration strategy had the highest level of resilience. In contrast, those who adopted the marginalization strategy had the lowest level of resilience. Those using the assimilation strategy were in the second place, with the separation strategy in the third place [67].

## Discussion

With 3,000 years of history behind them, China’s Qiang ethnic group has attracted much attention in recent times because of the earthquake in Sichuan, which occurred on May 12, 2008. The earthquake caused an enormous disaster, which was not only a natural calamity, but also a cultural catastrophe. Many precious Qiang cultural relics were destroyed, and some of the Qiang intangible cultural heritage inheritance of the people was lost in the earthquake. Thus, it is important to understand how to preserve the Qiang ethnic heritage culture and their ways of acculturating after this disaster, and also to understand the challenges faced by them in recovering their mental health. The current research sought to describe and explain the relationship between acculturation and resilience of the Qiang people.

Resilience from a cross-cultural perspective involves the examination of multiple phenomena, including individual development, community impact, and cultural systems of thought. The Variable-focused approaches to resilience research focus on the relationships among stress /adversity, the influencing factors of resilience and psychosocial functions. In this study, the structural equation model of resilience showed that personality and spiritual belief had a mediating effect on resilience, and perceived social support play a moderation effect after the 2008 Chinese earthquake. It was found that 898 Qiang people’s integration acculturation strategy could positively predict resilience; these results are consistent with the previous research [68–70].

### The Mediating Effect of Personality and Spiritual Belief

Intercultural psychology has shown that cultural groups and their individual members (both immigrants and ethnic groups) usually undergo cultural and psychological changes following intercultural contact [10, 11]. Retaining norms and values of their original culture is associated with their lower PTSD-symptomatology. In the current study, Qiang cultural identity and National cultural identity were two main cultural identities in Qiang ethnicity.

The correlational analysis (Table 1) indicated that Qiang’s two cultural identities were positively related (.44). This indicates that the Qiang people were able to identify with both cultures, and both the identities were positively related to personality, spiritual beliefs, social support, and resilience. Particularly important were the positive correlations between resilience and Qiang identity (.30) and National identity (.42), showing that both identities had a positive impact on resilience. Other correlations ranged from .49 for resilience and personality to .13 for resilience and social support. These findings correspond to recent research by Ikizer [71] who found that religion, health, and positive personality characteristics were the most pronounced factors that were perceived by survivors as being associated with resilience.

The conceptual model (Fig 1) was based on previous research on resilience that showed that personality and spiritual belief could mediate the effect on cultural identities and resilience. In order to verify this conceptual model, we introduced all variables into the modified model. Fig 2 clearly shows the paths of the effect of personality on cultural identities and resilience (the path *a*_*1*_, *b*_*1*_, and *d*_*1*_), and the effect of spiritual belief on resilience (the path *a*_*2*_, *b*_*2*_, and *d*_*2*_). Moreover, the role of the two cultural identities on resilience displayed significant changes after combining with personality or spiritual belief. That is, personality and spiritual beliefs had mediating effects on cultural identities and resilience. In essence, the model pertains to protective factor model. The stressors impacted Qiang people’s cultural identities and disrupted their state of resilience. The personality traits and spiritual beliefs buffered the exposure to extreme stress and promoted resilience recovery. Acculturation is not a unidimensional process [22]. People may acculturate towards the new culture while retaining their heritage culture [11]. Though Qiang people lived in mountains and valleys, they successfully integrated into the culture of the larger society with the development of economic and social ties with China. Qiang society has been gradually influenced by the dominant culture in daily life, social customs, and religion. This is evidenced by their mean score on NCI being higher than QCI (in Table 1).

While Qiang people strived to hold on to and cherish their ethnic culture, they also attempted to adapt to the Chinese society. Koenig and colleagues [72] have provided hypothetical causal models of the religion-health connection; religion and spirituality lead to some psychological traits such as forgiveness, self-discipline and patience. Moreover, spirituality beliefs served as an intrapersonal factor associated with social adaptation and were a coping resource [73]. This was manifested through trust and faith, and by promoting individual strength to sustain the hope for a future [74].

Moreover, Qiang people’s personality traits such as extraversion (e.g., they are keen on dancing and singing) and openness (Qiang people are hospitable people) could promote their integration into the dominant Chinese culture and society. In a meta-analysis of the role of integration in adaptation during acculturation, this double identity and the acculturation strategy of integration have been shown to help individuals adapt while living interculturally [75].

Recent research has indicated that immigrant’s personality traits were closely related to their resilience [76]. Resilience was associated with a personality trait pattern that is cooperative, responsible, optimistic, and mature [77]. Interactions among different combinations of personality traits have strong effects on the perception of mental health [78]. In our study, Qiang people’s two cultural identities jointly contributed to 33.11% of the variance being accounted for resilience through personality and spiritual beliefs. In addition, Fig 2 also presented the direct effects of two cultural identities on resilience (the path *c*_*1*_, *c*_*2*_), and spiritual belief’s effect on personality (the path *f*). All the path coefficients of validation model were good fit for the data (in Table 2). These results supported the view that personality traits and spiritual beliefs can be subject to cultural influence [79] and predict on resilience [47, 80].

### The Moderating Effect of Social Support

Social support refers to the resources provided by others that assist individual in daily activities [81]. Higher levels of perceived social support are known to decrease negative psychological outcomes by providing a buffer from stressful events [82].

After the Wenchuan earthquake, the Chinese government provided a large number of resources for positive coping, such as rebuilding houses, public facilities and roads to recover from adversity as soon as possible. Many non-governmental organizations also played a large role in the reconstruction of their community and society. There were many temporary tent schools set up by volunteers in the earthquake zone. Some important donations from all over the world through non-governmental organizations were transported to the disaster area. Meanwhile, the Qiang people took some positive self-help measures in daily life to cope with the adversity caused by the disaster adversity. In this process, Qiang’s heritage cultures and customs attracted unprecedented attention of the public, and Qiang people got additional social support s including sufficient relief materials rebuilding of many of the post-disaster rebuilding projects by the state and local government, and a lot of spiritual encouragement and emotional supports from family and friends. Moreover, optimism, self-confidence, and persistence, as the invisible power, existed in Qiang ethnic culture, and this helped people cope with the distress and adversity.

However, an important question here is whether these sources of social support influenced the relationship of cultural identities with resilience. We supposed that there was a moderating effect on cultural identities and resilience through perceived social support. Table 3 verified this assumption: the result showed that there was a significant difference in prediction effect on cultural identity and resilience under the high or low level of social support or low. Furthermore, the interaction analysis verified that the effect of cultural identities was effect on resilience was affected by high or low levels of social support; that is, social support moderated the path of cultural identity to resilience. Research (e.g., [83]) has shown that social support can be a robust protective factor when people experience stressful events. Often people can rely on friends and family for support, they can rely on their own strengths or seek meaning in religious and spiritual beliefs [84]. In the present study, perceived social support from family, friends and important others (colleagues, government or nongovernment organizations) became the main source of support among Qiang people. To sum up, it was inferred that resilience was promoted via a number of protective factors linked to lower stress, including a strong sense of spirituality belief and perceived social support [85, 86]. Moreover, perceived social support moderated the path of cultural identity to resilience of Qiang people after the earthquake.

### Integration is the Optimum Acculturation Strategy for Qiang People’s Resilience

Berry [12] proposed that there were four acculturation strategies based on peoples’ orientation to two underlying dimensions. In this study, these two dimensions were conceptualized and operationalized as Qiang cultural identity (QCI) and National cultural identity (NCI). When crossed, they produced four acculturation strategies of integration, assimilation, separation and marginalization. A one-way analysis on cultural identities and resilience indicated that there were significant differences in resilience across all four acculturation strategies, and the acculturation strategies could significantly affect Qiang’s resilience. The integration strategy (about 38% of the sample) was the most optimal style to promote the development of resilience, followed by assimilation (about 21%), separation (about 11%) and finally marginalization (about 30%). As expected, the integration strategy was the most the preferred style by Qiang sample, indicating that multicultural values were accepted by them which is consistent with the Chinese policy of national unity. Furthermore, Qiang people may not have suffered intense psychological conflict when they attempted to integrate into dominant cultural society This result is consistent with many other findings (e.g., [12, 68, 69, 75, 87]. A recent study has found that low assimilation individuals were more likely to experience negative life events, and also more likely to experience post-disaster panic attacks, and have higher anxiety, and have poorer mental health status [23].

## Conclusion

Resilience is the ability to spring back from adversity and to successfully adapt after traumatic events. The present study verified that two cultural identities and four acculturation strategies (based on ethnic and National cultural identities) could significantly predict on resilience in people of Qiang ethnicity. The structural equation model analysis also showed that personality and spiritual beliefs had a mediating effect on resilience, and social support moderated the path of cultural identities to resilience. Perhaps the most significant contribution of this study is that the Qiang peoples’ cultural identities and their acculturation strategies were able to predict their resilience after the devastation of the 2008 Sichuan earthquake.

There are some limitations to the present study. First, the research is largely based on quantitative methods. In future studies, it would be illuminating to combine quantitative and qualitative methods in order to explore the meaning of the relationship between acculturation and resilience. Second, the four acculturation strategies can be measured in various ways, including using scales to directly measure the four strategies. In the present study we used an indirect measure and inferred the four strategies from the two cultural identities. In future studies, cross-validation could be achieved by using both kinds of measurement.

## Supporting Information

S1 Dataset SPSS data file showing raw variables data and related information for the participants. (SAV)
